# Bis(μ-5-diisopropyl­amino-1,2,3,4-tetra­zolido-κ^2^ *N* ^2^:*N* ^3^)bis­[(triisopropyl­phosphane)copper(I)]

**DOI:** 10.1107/S1600536811022719

**Published:** 2011-06-25

**Authors:** Issam Kobrsi, Ghada Bassioni

**Affiliations:** a Department of Chemistry, The Petroleum Institute, PO Box 2533, Abu Dhabi, United Arab Emirates; b Chemical Engineering Program, The Petroleum Institute, PO Box 2533, Abu Dhabi, United Arab Emirates

## Abstract

In the binuclear centrosymmetric crystal structure of the title compound, [Cu_2_(C_7_H_14_N_5_)_2_(C_9_H_21_P)_2_], all atoms except those of the isopropyl groups are approximately co-planar. The Cu(II) atom is in a distorted trigonal–planar CuN_2_P coordination. Bond angles around the amino N atom suggest *sp* ^2^ hybridization. Several intra­molecular C—H⋯N inter­actions are present involving tetra­zolate N atoms.

## Related literature

For background to the coordination chemistry of anionic five-membered nitro­gen-containing heterocyclic ligands, see: Nief (2001 ▶); Rottger *et al.* (1994 ▶); Hitzbleck *et al.* (2004 ▶); Gust *et al.* (2001 ▶, 2002 ▶); Dezelah *et al.* (2004 ▶); Sebe *et al.* (2005 ▶); Vela *et al.* (2006 ▶). Complexes containing these ligands have a strong tendency to form oligomeric and polymeric structures, see: Haasnoot (2000 ▶); Zhang *et al.* (2006 ▶); Dinca *et al.* (2006 ▶). η^1^ Coordination is the most commonly observed binding mode in monomeric complexes containing 1,2,4-triazolato and tetra­zolato ligands, see: Hunyh *et al.* (2003 ▶); Jiang *et al.* (2004 ▶). Theoretical predictions regarding the high stability of the penta­zolate (N_5_^−^) ion suggest that metal complexes containing this ligand might be stable enough to allow isolation, see: Frunzke *et al.* (2002 ▶); Lein *et al.* (2001 ▶); Burke *et al.* (2001 ▶). For our work on the synthesis, structures and mol­ecular orbital calculations of a series of Ba(alkyl­tetra­zol­ate)_2_(18-crown-6), K(alkyl­tetra­zolate)(18-crown-6), Ba(pen­ta­zolate)_2_(18-crown-6) and K(penta­zolate)(18-crown-6) complexes, which exhibited highly distorted tetra­zolato and penta­zolato ligand bonding, see: Kobrsi *et al.* (2005 ▶, 2006 ▶). For van der Waals radii, see: Allinger *et al.* (1968 ▶); Bondi (1964 ▶). 
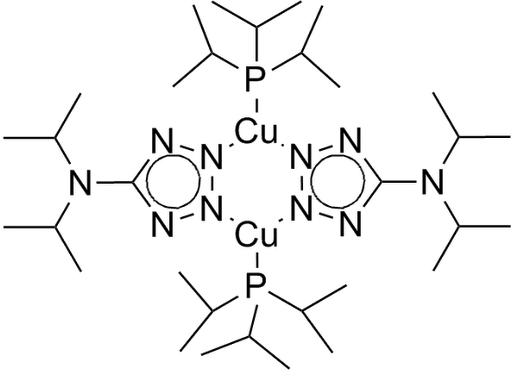


## Experimental

### 

#### Crystal data

[Cu_2_(C_7_H_14_N_5_)_2_(C_9_H_21_P)_2_]
*M* *_r_* = 784.02
Triclinic, 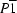

*a* = 7.3573 (6) Å
*b* = 10.8987 (8) Å
*c* = 12.7134 (9) Å
α = 94.273 (2)°
β = 96.993 (2)°
γ = 93.548 (2)°
*V* = 1006.43 (13) Å^3^
*Z* = 1
Mo *K*α radiation
μ = 1.17 mm^−1^
*T* = 100 K
0.37 × 0.28 × 0.21 mm

#### Data collection

Bruker APEXII diffractometer
Absorption correction: multi-scan (*SADABS*; Bruker, 2005 ▶) *T*_min_ = 0.675, *T*_max_ = 0.791
17280 measured reflections
4689 independent reflections
4336 reflections with *I* > 2σ(*I*)
*R*_int_ = 0.042

#### Refinement

*R*[*F* ^2^ > 2σ(*F* ^2^)] = 0.029
*wR*(*F* ^2^) = 0.079
*S* = 1.05
4689 reflections
218 parameters
H-atom parameters constrained
Δρ_max_ = 0.70 e Å^−3^
Δρ_min_ = −0.42 e Å^−3^

### 

Data collection: *APEX2* (Bruker, 2005 ▶); cell refinement: *SAINT-Plus* (Bruker, 2005 ▶); data reduction: *SAINT-Plus*; program(s) used to solve structure: *SHELXS97* (Sheldrick, 2008 ▶); program(s) used to refine structure: *SHELXL97* (Sheldrick, 2008 ▶); molecular graphics: *SHELXTL-Plus* (Sheldrick, 2008 ▶); software used to prepare material for publication: *SHELXTL-Plus* (Sheldrick, 2008 ▶).

## Supplementary Material

Crystal structure: contains datablock(s) global, I. DOI: 10.1107/S1600536811022719/hp2003sup1.cif

Structure factors: contains datablock(s) I. DOI: 10.1107/S1600536811022719/hp2003Isup2.hkl

Additional supplementary materials:  crystallographic information; 3D view; checkCIF report

## Figures and Tables

**Table:** 

Cu1—P1	2.1957 (5)
Cu1—N2	1.9919 (14)
Cu1—N3	1.9938 (13)

**Table:** 

P1—Cu1—N2	126.53 (4)
P1—Cu1—N3	126.52 (4)
N2—Cu1—N3	106.96 (5)

**Table 2:** Hydrogen-bond geometry (Å, °)

*D*—H⋯*A*	*D*—H	H⋯*A*	*D*⋯*A*	*D*—H⋯*A*
C3—H3*A*⋯N4^i^	0.98	2.58	3.182 (2)	119
C4—H4*B*⋯N4^i^	0.98	2.48	3.082 (2)	120
C5—H5⋯N1	1.00	2.32	2.784 (2)	107

Symmetry code: (i) 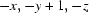.

**Fig. 1.**
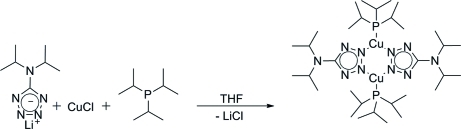
Reaction scheme for the preparation of the title compound.

**Fig. 2.**
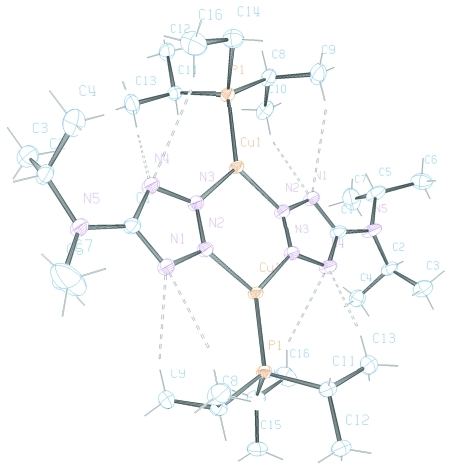
A perspective view of title compound showing the labelling of the non-H atoms. Thermal ellipsoids are shown at 50% probability levels, except for H atoms.

## supplementary crystallographic information

### Comment

The coordination chemistry of anionic five-membered nitrogen heterocyclic ligands has generated considerable recent interest from several different perspectives (Nief 2001, Rottger *et al.* 1994, Hitzbleck *et al.* 2004, Gust *et al.* 2001, Dezelah *et al.* 2004, Sebe *et al.* 2005, Gust *et al.* 2002, Vela *et al.* 2006). Due to the presence of many nitrogen atoms in 1,2,4-triazolato and tetrazolato ligands, complexes containing these ligands have a strong tendency to form oligomeric and polymeric compounds through bridging ligand coordination modes (Haasnoot 2000, Zhang *et al.* 2006, Dinca *et al.* 2006). Furthermore, η^1^-coordination is the most commonly observed binding mode in monomeric complexes containing 1,2,4-triazolato and tetrazolato ligands (Jiang *et al.* 2004, Hunyh *et al.* 2003). Theoretical predictions regarding the high stability of the pentazolate (N_5_^-^) ion suggest that metal complexes containing this ligand might be stable enough to allow isolation (Frunzke *et al.* 2002, Lein *et al.* 2001, Burke *et al.* 2001).

Since complexes containing N_5_^-^ ligands may be at the edge of isolability due to facile loss of dinitrogen, it is important to develop a knowledge base that allows the synthesis of soluble, tractable 1,2,4-triazolato and tetrazolato complexes. Presumably, the basic coordination chemistry of pentazolato ligands will share similarities with that of tetrazolato ligands.

Several years ago, we reported the synthesis, structure, and molecular orbital calculations of a series of barium complexes of the formula Ba(alkyltetrazolate)_2_(18-crown-6), potassium complexes of formula *K*(alkyltetrazolate)(18-crown-6), as well as calculations of Ba(pentazolate)_2_(18-crown-6) and K(pentazolate)(18-crown-6). These complexes contained highly distorted tetrazolato and pentazolato ligand bonding (Kobrsi *et al.* 2005, Kobrsi *et al.* 2006).

The present work demonstrates the stabilization of copper tetrazolate complexes using a 2-electron donor phosphane ligand. The copper complex crystallizes as a dimer having all nuclei except the isopropyl groups' in the same plane. The phosphane ligands are terminal, while each tetrazolate ligand bridges two Cu(I) centers.

While the work aimed for a monomeric complex, it can be concluded that the combination of isopropyl groups in the phosphane ligand and the tetrazolate ligand does not provide the necessary steric repulsion. However, enough steric hindrance is provided for the tetrazolate to coordinate in an N2—N3 bridging mode as opposed to the normally observed N1—N2 bridging mode.

The C1—N5—C5 angle of 120.30 (13)° and the C2—N5—C5 angle of 118.89 (13)° suggest that the N-atom of the amino group is *sp*^2^-hybridized, having its electrons donated to the aromatic ring, thus providing stability to the electron-deficient heterocycle.

Several intramolecular CH—N interactions exist between the tetrazolate's N1 and N4, and the hydrogen atoms on C9, C10, C13, C16. The CH—N distances range from 2.64 to 2.77 Å, whereas the sum of the van der Waals radii for N and H is about 2.7–3.0 Å (Bondi 1964, Allinger *et al.* 1968), which supports weak, attractive CH—N interactions. These types of interactions have been previously observed, where calculations have shown that these interactions provide stability to the heterocycle (Kobrsi *et al.* 2005, Kobrsi *et al.* 2006).

### Experimental

A 100 ml Schlenk flask was charged with copper(I) chloride (0.300 g, 3.06 mmol), 40 ml of THF, and a stir bar under an inert atmosphere of argon. Triisopropylphosphane (0.491 g, 3.06 mmol) was added to the mixture while stirring. After 2 h, lithium 5-diisopropylamino-1,2,3,4-tetrazolate (0.536 g, 3.06 mmol) was added, and the reaction mixture was allowed to stir for 18 h at room temperature. The solvent was then removed under vacuum, the products extracted in 30 ml of hexane, and the resulting mixture filtered through a pad of celite. Single crystals were grown from a supersaturated solution at 0°C in the form of white needles. Crystalline samples were mounted in sealed thin wall capillaries under nitrogen atmosphere for X-ray data collection.

### Crystal data

**Table:**

[Cu_2_(C_7_H_14_N_5_)_2_(C_9_H_21_P)_2_]	*Z* = 1
*M**_r_* = 784.02	*F*(000) = 420
Triclinic, *P*1	*D*_x_ = 1.294 Mg m^−^^3^
Hall symbol: -P 1	Mo *K*α radiation, λ = 0.71073 Å
*a* = 7.3573 (6) Å	Cell parameters from 8161 reflections
*b* = 10.8987 (8) Å	θ = 2.8–28.2°
*c* = 12.7134 (9) Å	µ = 1.17 mm^−^^1^
α = 94.273 (2)°	*T* = 100 K
β = 96.993 (2)°	Fragment, colorless
γ = 93.548 (2)°	0.37 × 0.28 × 0.21 mm
*V* = 1006.43 (13) Å^3^	

### Data collection

**Table:**

Bruker APEXII diffractometer	4689 independent reflections
Radiation source: fine-focus sealed tube	4336 reflections with *I* > 2σ(*I*)
graphite	*R*_int_ = 0.042
Bruker APEX2 scans	θ_max_ = 28.2°, θ_min_ = 3.1°
Absorption correction: multi-scan (*SADABS*; Bruker, 2005)	*h* = −9→9
*T*_min_ = 0.675, *T*_max_ = 0.791	*k* = −14→14
17280 measured reflections	*l* = 0→16

### Refinement

**Table:**

Refinement on *F*^2^	Primary atom site location: structure-invariant direct methods
Least-squares matrix: full	Secondary atom site location: difference Fourier map
*R*[*F*^2^ > 2σ(*F*^2^)] = 0.029	Hydrogen site location: inferred from neighbouring sites
*wR*(*F*^2^) = 0.079	H-atom parameters constrained
*S* = 1.05	*w* = 1/[σ^2^(*F*_o_^2^) + (0.0402*P*)^2^ + 0.4118*P*] where *P* = (*F*_o_^2^ + 2*F*_c_^2^)/3
4689 reflections	(Δ/σ)_max_ = 0.001
218 parameters	Δρ_max_ = 0.70 e Å^−^^3^
0 restraints	Δρ_min_ = −0.42 e Å^−^^3^

### Special details

**Table:**

Geometry. All e.s.d.'s (except the e.s.d. in the dihedral angle between two l.s. planes) are estimated using the full covariance matrix. The cell e.s.d.'s are taken into account individually in the estimation of e.s.d.'s in distances, angles and torsion angles; correlations between e.s.d.'s in cell parameters are only used when they are defined by crystal symmetry. An approximate (isotropic) treatment of cell e.s.d.'s is used for estimating e.s.d.'s involving l.s. planes.
Refinement. Refinement of *F*^2^ against ALL reflections. The weighted *R*-factor *wR* and goodness of fit *S* are based on *F*^2^, conventional *R*-factors *R* are based on *F*, with *F* set to zero for negative *F*^2^. The threshold expression of *F*^2^ > σ(*F*^2^) is used only for calculating *R*-factors(gt) *etc*. and is not relevant to the choice of reflections for refinement. *R*-factors based on *F*^2^ are statistically about twice as large as those based on *F*, and *R*- factors based on ALL data will be even larger.

### Fractional atomic coordinates and isotropic or equivalent isotropic displacement parameters (Å^2^)

**Table:**

	*x*	*y*	*z*	*U*_iso_*/*U*_eq_	
Cu1	0.17941 (2)	0.40943 (2)	0.06357 (1)	0.0175 (1)	
P1	0.40113 (5)	0.30668 (4)	0.13812 (3)	0.0178 (1)	
N1	0.11874 (19)	0.59579 (13)	0.23257 (10)	0.0229 (4)	
N2	0.06116 (17)	0.54829 (13)	0.13256 (10)	0.0204 (3)	
N3	0.05711 (17)	0.38081 (13)	−0.08583 (10)	0.0200 (4)	
N4	0.08031 (19)	0.28290 (13)	−0.15304 (10)	0.0245 (4)	
N5	0.0599 (2)	0.78110 (15)	0.33143 (11)	0.0314 (4)	
C1	0.0307 (2)	0.69968 (15)	0.24179 (12)	0.0231 (4)	
C2	−0.0740 (2)	0.87242 (15)	0.35075 (12)	0.0238 (4)	
C3	−0.0506 (2)	0.98014 (17)	0.28292 (14)	0.0302 (5)	
C4	−0.2723 (2)	0.81977 (18)	0.33976 (14)	0.0316 (5)	
C5	0.1839 (3)	0.74562 (18)	0.42230 (14)	0.0354 (5)	
C6	0.2998 (2)	0.8553 (2)	0.47853 (14)	0.0341 (5)	
C7	0.0810 (4)	0.6751 (2)	0.4980 (2)	0.0591 (8)	
C8	0.4926 (2)	0.36010 (15)	0.27701 (12)	0.0246 (4)	
C9	0.5791 (3)	0.49258 (17)	0.28358 (15)	0.0335 (5)	
C10	0.3424 (3)	0.35205 (18)	0.34983 (13)	0.0324 (5)	
C11	0.3136 (2)	0.14523 (15)	0.14860 (13)	0.0227 (4)	
C12	0.4392 (2)	0.06264 (16)	0.21261 (14)	0.0265 (5)	
C13	0.2369 (3)	0.07915 (17)	0.04102 (14)	0.0315 (5)	
C14	0.6088 (2)	0.31413 (17)	0.06842 (13)	0.0261 (5)	
C15	0.7777 (2)	0.25545 (18)	0.11955 (15)	0.0300 (5)	
C16	0.5643 (2)	0.2698 (2)	−0.04897 (14)	0.0360 (6)	
H2	−0.04210	0.90660	0.42640	0.0290*	
H3A	−0.07590	0.95060	0.20750	0.0450*	
H3B	0.07550	1.01720	0.29820	0.0450*	
H3C	−0.13640	1.04200	0.29930	0.0450*	
H4A	−0.28240	0.75290	0.38670	0.0470*	
H4B	−0.31160	0.78760	0.26590	0.0470*	
H4C	−0.35080	0.88490	0.35960	0.0470*	
H5	0.26960	0.68830	0.39280	0.0420*	
H6A	0.22370	0.90680	0.51910	0.0510*	
H6B	0.35190	0.90370	0.42600	0.0510*	
H6C	0.39940	0.82690	0.52710	0.0510*	
H7A	0.00340	0.60680	0.45800	0.0890*	
H7B	0.00410	0.73060	0.53400	0.0890*	
H7C	0.16930	0.64230	0.55100	0.0890*	
H8	0.59030	0.30530	0.30200	0.0290*	
H9A	0.62420	0.52010	0.35780	0.0500*	
H9B	0.48670	0.54700	0.25600	0.0500*	
H9C	0.68160	0.49530	0.24110	0.0500*	
H10A	0.24340	0.40320	0.32520	0.0490*	
H10B	0.39400	0.38160	0.42270	0.0490*	
H10C	0.29350	0.26620	0.34840	0.0490*	
H11	0.20500	0.15240	0.18830	0.0270*	
H12A	0.54300	0.04410	0.17400	0.0400*	
H12B	0.36980	−0.01440	0.22250	0.0400*	
H12C	0.48510	0.10550	0.28220	0.0400*	
H13A	0.16230	0.00450	0.05180	0.0470*	
H13B	0.33840	0.05640	0.00200	0.0470*	
H13C	0.16050	0.13400	0.00010	0.0470*	
H14	0.64690	0.40400	0.06980	0.0310*	
H15A	0.88080	0.27150	0.07930	0.0450*	
H15B	0.75040	0.16620	0.11910	0.0450*	
H15C	0.80990	0.29100	0.19310	0.0450*	
H16A	0.54690	0.17950	−0.05660	0.0540*	
H16B	0.66580	0.29660	−0.08710	0.0540*	
H16C	0.45150	0.30470	−0.07870	0.0540*	

### Atomic displacement parameters (Å^2^)

**Table:**

	*U*^11^	*U*^22^	*U*^33^	*U*^12^	*U*^13^	*U*^23^
Cu1	0.0202 (1)	0.0177 (1)	0.0138 (1)	0.0075 (1)	−0.0031 (1)	−0.0010 (1)
P1	0.0195 (2)	0.0181 (2)	0.0149 (2)	0.0060 (1)	−0.0031 (1)	0.0007 (1)
N1	0.0288 (7)	0.0237 (7)	0.0147 (6)	0.0109 (5)	−0.0040 (5)	−0.0037 (5)
N2	0.0243 (6)	0.0209 (7)	0.0150 (5)	0.0076 (5)	−0.0023 (4)	−0.0015 (5)
N3	0.0225 (6)	0.0214 (7)	0.0153 (6)	0.0070 (5)	−0.0014 (4)	−0.0024 (5)
N4	0.0302 (7)	0.0263 (7)	0.0158 (6)	0.0133 (5)	−0.0039 (5)	−0.0042 (5)
N5	0.0422 (8)	0.0316 (8)	0.0179 (6)	0.0223 (6)	−0.0099 (6)	−0.0082 (6)
C1	0.0266 (7)	0.0243 (8)	0.0175 (7)	0.0105 (6)	−0.0033 (5)	−0.0021 (6)
C2	0.0309 (8)	0.0226 (8)	0.0176 (7)	0.0116 (6)	0.0002 (6)	−0.0031 (6)
C3	0.0348 (9)	0.0294 (9)	0.0275 (8)	0.0085 (7)	0.0046 (7)	0.0028 (7)
C4	0.0366 (9)	0.0285 (9)	0.0295 (8)	0.0031 (7)	0.0058 (7)	−0.0022 (7)
C5	0.0477 (10)	0.0324 (10)	0.0223 (8)	0.0216 (8)	−0.0142 (7)	−0.0080 (7)
C6	0.0295 (8)	0.0473 (12)	0.0243 (8)	0.0077 (8)	−0.0017 (6)	0.0003 (8)
C7	0.0697 (15)	0.0431 (13)	0.0551 (14)	−0.0126 (11)	−0.0350 (12)	0.0262 (11)
C8	0.0310 (8)	0.0216 (8)	0.0186 (7)	0.0077 (6)	−0.0087 (6)	−0.0002 (6)
C9	0.0390 (9)	0.0245 (9)	0.0319 (9)	0.0043 (7)	−0.0131 (7)	−0.0039 (7)
C10	0.0497 (10)	0.0301 (10)	0.0178 (7)	0.0138 (8)	0.0008 (7)	0.0009 (7)
C11	0.0234 (7)	0.0193 (7)	0.0248 (7)	0.0056 (6)	−0.0015 (6)	0.0016 (6)
C12	0.0301 (8)	0.0206 (8)	0.0280 (8)	0.0069 (6)	−0.0038 (6)	0.0041 (6)
C13	0.0355 (9)	0.0228 (9)	0.0320 (9)	0.0012 (7)	−0.0108 (7)	−0.0006 (7)
C14	0.0231 (7)	0.0303 (9)	0.0252 (8)	0.0057 (6)	0.0005 (6)	0.0047 (7)
C15	0.0213 (7)	0.0355 (10)	0.0344 (9)	0.0079 (7)	0.0012 (6)	0.0089 (7)
C16	0.0294 (8)	0.0539 (13)	0.0250 (8)	0.0029 (8)	0.0058 (7)	0.0023 (8)

### Geometric parameters (Å, °)

**Table:**

Cu1—P1	2.1957 (5)	C4—H4C	0.9800
Cu1—N2	1.9919 (14)	C5—H5	1.0000
Cu1—N3	1.9938 (13)	C6—H6A	0.9800
P1—C8	1.8490 (16)	C6—H6B	0.9800
P1—C11	1.8559 (17)	C6—H6C	0.9800
P1—C14	1.8578 (16)	C7—H7A	0.9800
N1—N2	1.3445 (18)	C7—H7B	0.9800
N1—C1	1.343 (2)	C7—H7C	0.9800
N2—N3^i^	1.3164 (19)	C8—H8	1.0000
N3—N4	1.3491 (19)	C9—H9A	0.9800
N4—C1^i^	1.343 (2)	C9—H9B	0.9800
N5—C1	1.378 (2)	C9—H9C	0.9800
N5—C2	1.472 (2)	C10—H10A	0.9800
N5—C5	1.473 (2)	C10—H10B	0.9800
C2—C3	1.521 (2)	C10—H10C	0.9800
C2—C4	1.521 (2)	C11—H11	1.0000
C5—C6	1.505 (3)	C12—H12A	0.9800
C5—C7	1.519 (3)	C12—H12B	0.9800
C8—C9	1.534 (3)	C12—H12C	0.9800
C8—C10	1.528 (3)	C13—H13A	0.9800
C11—C12	1.535 (2)	C13—H13B	0.9800
C11—C13	1.524 (2)	C13—H13C	0.9800
C14—C15	1.530 (2)	C14—H14	1.0000
C14—C16	1.524 (2)	C15—H15A	0.9800
C2—H2	1.0000	C15—H15B	0.9800
C3—H3A	0.9800	C15—H15C	0.9800
C3—H3B	0.9800	C16—H16A	0.9800
C3—H3C	0.9800	C16—H16B	0.9800
C4—H4A	0.9800	C16—H16C	0.9800
C4—H4B	0.9800		
			
Cu1···H15A^ii^	2.6200	H5···N1	2.3200
N1···N4^i^	2.235 (2)	H6A···C2	2.8600
N2···N3	3.2030 (19)	H6A···H2	2.1600
N2···N4^i^	2.184 (2)	H6A···H7B	2.4700
N3···N2	3.2030 (19)	H6A···H2^viii^	2.6000
N3···N1^i^	2.1779 (18)	H6A···H3C^viii^	2.5000
N4···C4^i^	3.082 (2)	H6B···H4C^v^	2.4500
N4···C3^i^	3.182 (2)	H6C···H12C^ix^	2.5100
N4···N1^i^	2.235 (2)	H7A···C1	3.0200
N1···H10A	2.6400	H7B···C2	2.9100
N1···H9B	2.7800	H7B···H2	2.4500
N1···H5	2.3200	H7B···H6A	2.4700
N1···H16B^iii^	2.8500	H7C···H8^ix^	2.4200
N2···H16B^iii^	2.6900	H8···C12	2.9000
N3···H15A^ii^	2.8900	H8···C15	2.8700
N4···H13C	2.6600	H8···H12C	2.2500
N4···H16C	2.7700	H8···H15C	2.2600
N4···H3A^i^	2.5800	H8···H7C^ix^	2.4200
N4···H4B^i^	2.4800	H9A···H4A^v^	2.5700
C3···N4^i^	3.182 (2)	H9A···H10B	2.4600
C4···N4^i^	3.082 (2)	H9B···N1	2.7800
C12···C15	3.530 (2)	H9B···H10A	2.5800
C13···C16	3.440 (3)	H9C···C14	2.8200
C15···C12	3.530 (2)	H9C···H14	2.3000
C16···C13	3.440 (3)	H9C···H15C	2.5300
C1···H7A	3.0200	H10A···N1	2.6400
C1···H4B	2.7900	H10A···H9B	2.5800
C1···H3A	2.9400	H10B···H9A	2.4600
C2···H7B	2.9100	H10C···C11	2.8000
C2···H6A	2.8600	H10C···C12	3.0400
C3···H11^iv^	2.9900	H10C···H11	2.3000
C6···H2	2.6300	H10C···H12C	2.4800
C7···H2	2.9000	H11···C3^vi^	2.9900
C7···H4A	3.0700	H11···C10	2.9200
C8···H15C	2.8000	H11···H3B^vi^	2.3400
C8···H12C	2.7800	H11···H10C	2.3000
C9···H14	2.9300	H12A···C15	2.9700
C9···H15C	3.1000	H12A···H13B	2.5200
C9···H4A^v^	3.0900	H12A···H15B	2.1800
C10···H11	2.9200	H12B···H3B^vi^	2.5100
C10···H12C	3.0500	H12B···H13A	2.5300
C11···H3B^vi^	3.0900	H12C···C8	2.7800
C11···H10C	2.8000	H12C···C10	3.0500
C12···H3B^vi^	3.0400	H12C···H8	2.2500
C12···H15B	2.9100	H12C···H10C	2.4800
C12···H10C	3.0400	H12C···H6C^ix^	2.5100
C12···H8	2.9000	H13A···H12B	2.5300
C13···H13A^vii^	3.0800	H13A···C13^vii^	3.0800
C13···H16A	2.9200	H13A···H13A^vii^	2.5800
C14···H9C	2.8200	H13B···C16	2.9300
C15···H8	2.8700	H13B···H12A	2.5200
C15···H12A	2.9700	H13B···H16A	2.2100
C16···H13B	2.9300	H13C···N4	2.6600
H2···C6	2.6300	H14···C9	2.9300
H2···C7	2.9000	H14···H9C	2.3000
H2···H6A	2.1600	H15A···Cu1^v^	2.6200
H2···H7B	2.4500	H15A···N3^v^	2.8900
H2···H6A^viii^	2.6000	H15A···H16B	2.5300
H3A···C1	2.9400	H15B···C12	2.9100
H3A···N4^i^	2.5800	H15B···H12A	2.1800
H3B···C11^iv^	3.0900	H15B···H16A	2.5500
H3B···C12^iv^	3.0400	H15C···C8	2.8000
H3B···H11^iv^	2.3400	H15C···C9	3.1000
H3B···H12B^iv^	2.5100	H15C···H8	2.2600
H3C···H4C	2.4800	H15C···H9C	2.5300
H3C···H6A^viii^	2.5000	H16A···C13	2.9200
H4A···C7	3.0700	H16A···H13B	2.2100
H4A···C9^ii^	3.0900	H16A···H15B	2.5500
H4A···H9A^ii^	2.5700	H16B···H15A	2.5300
H4B···C1	2.7900	H16B···N1^iii^	2.8500
H4B···N4^i^	2.4800	H16B···N2^iii^	2.6900
H4B···H16C^i^	2.5800	H16C···N4	2.7700
H4C···H3C	2.4800	H16C···H4B^i^	2.5800
H4C···H6B^ii^	2.4500		
			
P1—Cu1—N2	126.53 (4)	C5—C6—H6B	109.00
P1—Cu1—N3	126.52 (4)	C5—C6—H6C	109.00
N2—Cu1—N3	106.96 (5)	H6A—C6—H6B	109.00
Cu1—P1—C8	116.09 (5)	H6A—C6—H6C	109.00
Cu1—P1—C11	109.53 (5)	H6B—C6—H6C	109.00
Cu1—P1—C14	112.78 (6)	C5—C7—H7A	110.00
C8—P1—C11	103.02 (7)	C5—C7—H7B	110.00
C8—P1—C14	103.05 (7)	C5—C7—H7C	109.00
C11—P1—C14	111.91 (8)	H7A—C7—H7B	109.00
N2—N1—C1	103.91 (12)	H7A—C7—H7C	109.00
Cu1—N2—N1	122.29 (10)	H7B—C7—H7C	109.00
Cu1—N2—N3^i^	126.91 (10)	P1—C8—H8	108.00
N1—N2—N3^i^	109.86 (13)	C9—C8—H8	108.00
Cu1—N3—N4	124.44 (10)	C10—C8—H8	108.00
Cu1—N3—N2^i^	125.54 (10)	C8—C9—H9A	110.00
N2^i^—N3—N4	110.00 (12)	C8—C9—H9B	109.00
N3—N4—C1^i^	103.64 (13)	C8—C9—H9C	109.00
C1—N5—C2	120.27 (13)	H9A—C9—H9B	109.00
C1—N5—C5	117.08 (15)	H9A—C9—H9C	109.00
C2—N5—C5	118.88 (14)	H9B—C9—H9C	109.00
N1—C1—N5	122.80 (14)	C8—C10—H10A	109.00
N1—C1—N4^i^	112.59 (14)	C8—C10—H10B	109.00
N4^i^—C1—N5	124.55 (15)	C8—C10—H10C	109.00
N5—C2—C3	110.45 (13)	H10A—C10—H10B	109.00
N5—C2—C4	114.69 (14)	H10A—C10—H10C	109.00
C3—C2—C4	112.02 (13)	H10B—C10—H10C	109.00
N5—C5—C6	111.51 (16)	P1—C11—H11	105.00
N5—C5—C7	111.98 (19)	C12—C11—H11	105.00
C6—C5—C7	112.07 (16)	C13—C11—H11	105.00
P1—C8—C9	110.57 (11)	C11—C12—H12A	109.00
P1—C8—C10	111.09 (11)	C11—C12—H12B	109.00
C9—C8—C10	110.22 (14)	C11—C12—H12C	109.00
P1—C11—C12	117.76 (11)	H12A—C12—H12B	109.00
P1—C11—C13	112.62 (12)	H12A—C12—H12C	109.00
C12—C11—C13	110.39 (14)	H12B—C12—H12C	109.00
P1—C14—C15	117.04 (12)	C11—C13—H13A	109.00
P1—C14—C16	111.77 (11)	C11—C13—H13B	110.00
C15—C14—C16	111.12 (14)	C11—C13—H13C	109.00
N5—C2—H2	106.00	H13A—C13—H13B	109.00
C3—C2—H2	106.00	H13A—C13—H13C	109.00
C4—C2—H2	106.00	H13B—C13—H13C	109.00
C2—C3—H3A	109.00	P1—C14—H14	105.00
C2—C3—H3B	109.00	C15—C14—H14	105.00
C2—C3—H3C	109.00	C16—C14—H14	105.00
H3A—C3—H3B	109.00	C14—C15—H15A	109.00
H3A—C3—H3C	109.00	C14—C15—H15B	109.00
H3B—C3—H3C	110.00	C14—C15—H15C	109.00
C2—C4—H4A	109.00	H15A—C15—H15B	110.00
C2—C4—H4B	109.00	H15A—C15—H15C	110.00
C2—C4—H4C	109.00	H15B—C15—H15C	109.00
H4A—C4—H4B	110.00	C14—C16—H16A	109.00
H4A—C4—H4C	109.00	C14—C16—H16B	109.00
H4B—C4—H4C	109.00	C14—C16—H16C	109.00
N5—C5—H5	107.00	H16A—C16—H16B	109.00
C6—C5—H5	107.00	H16A—C16—H16C	109.00
C7—C5—H5	107.00	H16B—C16—H16C	110.00
C5—C6—H6A	110.00		
			
N2—Cu1—P1—C8	−2.92 (8)	C8—P1—C14—C15	48.70 (15)
N2—Cu1—P1—C11	113.17 (7)	C8—P1—C14—C16	178.45 (13)
N2—Cu1—P1—C14	−121.49 (8)	C11—P1—C14—C15	−61.33 (15)
N3—Cu1—P1—C8	177.48 (7)	C11—P1—C14—C16	68.43 (15)
N3—Cu1—P1—C11	−66.43 (7)	C1—N1—N2—Cu1	168.56 (10)
N3—Cu1—P1—C14	58.91 (8)	C1—N1—N2—N3^i^	−1.07 (16)
P1—Cu1—N2—N1	4.13 (14)	N2—N1—C1—N5	−176.03 (14)
P1—Cu1—N2—N3^i^	171.90 (10)	N2—N1—C1—N4^i^	1.06 (18)
N3—Cu1—N2—N1	−176.21 (11)	Cu1—N2—N3^i^—Cu1^i^	9.93 (19)
N3—Cu1—N2—N3^i^	−8.43 (14)	Cu1—N2—N3^i^—N4^i^	−168.30 (10)
P1—Cu1—N3—N4	9.97 (14)	N1—N2—N3^i^—Cu1^i^	178.95 (10)
P1—Cu1—N3—N2^i^	−172.05 (10)	N1—N2—N3^i^—N4^i^	0.73 (17)
N2—Cu1—N3—N4	−169.69 (12)	Cu1—N3—N4—C1^i^	178.31 (10)
N2—Cu1—N3—N2^i^	8.29 (14)	N2^i^—N3—N4—C1^i^	0.06 (16)
Cu1—P1—C8—C9	−62.53 (13)	N3—N4—C1^i^—N1^i^	0.65 (17)
Cu1—P1—C8—C10	60.18 (13)	N3—N4—C1^i^—N5^i^	−176.39 (15)
C11—P1—C8—C9	177.79 (12)	C2—N5—C1—N1	−162.27 (15)
C11—P1—C8—C10	−59.51 (13)	C2—N5—C1—N4^i^	21.0 (2)
C14—P1—C8—C9	61.25 (14)	C5—N5—C1—N1	−4.4 (2)
C14—P1—C8—C10	−176.04 (12)	C5—N5—C1—N4^i^	178.88 (16)
Cu1—P1—C11—C12	−171.57 (10)	C1—N5—C2—C3	−78.37 (18)
Cu1—P1—C11—C13	58.21 (13)	C1—N5—C2—C4	49.4 (2)
C8—P1—C11—C12	−47.46 (13)	C5—N5—C2—C3	124.14 (16)
C8—P1—C11—C13	−177.68 (12)	C5—N5—C2—C4	−108.15 (18)
C14—P1—C11—C12	62.59 (14)	C1—N5—C5—C6	142.57 (15)
C14—P1—C11—C13	−67.63 (14)	C1—N5—C5—C7	−90.96 (19)
Cu1—P1—C14—C15	174.64 (11)	C2—N5—C5—C6	−59.2 (2)
Cu1—P1—C14—C16	−55.61 (14)	C2—N5—C5—C7	67.3 (2)

Symmetry codes: (i) −*x*, −*y*+1, −*z*; (ii) *x*−1, *y*, *z*; (iii) −*x*+1, −*y*+1, −*z*; (iv) *x*, *y*+1, *z*; (v) *x*+1, *y*, *z*; (vi) *x*, *y*−1, *z*; (vii) −*x*, −*y*, −*z*; (viii) −*x*, −*y*+2, −*z*+1; (ix) −*x*+1, −*y*+1, −*z*+1.

### Hydrogen-bond geometry (Å, °)

**Table:**

*D*—H···*A*	*D*—H	H···*A*	*D*···*A*	*D*—H···*A*
C3—H3A···N4^i^	0.98	2.58	3.182 (2)	119
C4—H4B···N4^i^	0.98	2.48	3.082 (2)	120
C5—H5···N1	1.00	2.32	2.784 (2)	107

Symmetry codes: (i) −*x*, −*y*+1, −*z*.
